# Interaction between Brain Natriuretic Peptide and QTc Prolongation on Mortality in Patients with Stroke-Heart Syndrome

**DOI:** 10.14336/AD.2025.10711

**Published:** 2026-07-31

**Authors:** Yongle Wang, Mingyi He, Lanjing Wang, Omar Elmadhoun, Fangyan Liu, Wenbo Zhao, Changhong Ren, Yuchuan Ding, Xunming Ji, Sijie Li

**Affiliations:** ^1^Department of Neurology, Xuanwu Hospital, Capital Medical University, Beijing, China.; ^2^Department of Emergency, Xuanwu Hospital, Capital Medical University, Beijing, China.; ^3^Division of Critical Care, Department of Anesthesiology and Perioperative Medicine, Mayo Clinic, Rochester, MN 55902, USA.; ^4^Beijing Key Laboratory of Hypoxic Conditioning Translational Medicine, Xuanwu Hospital, Capital Medical University, Beijing, China.; ^5^Department of Neurosurgery, Wayne State University School of Medicine, Detroit, MI 48201, USA.; ^6^Neuro Cardio Vascular Diseases Center, Xuanwu Hospital, Capital Medical University, Beijing, China.; ^7^Beijing Institute of Brain Disorders, Collaborative Innovation Center for Brain Disorders, Capital Medical University, Beijing, China.

**Keywords:** stroke, stroke-heart syndrome, brain natriuretic peptide, QTc prolongation

## Abstract

Stroke-heart syndrome (SHS) is associated with early mortality in patients with acute ischemic stroke (AIS). However, reliable methods for timely risk stratification remain elusive. Combined monitoring of neurohormonal activation and electrocardiography may help identifying early mortality risk in SHS patients. This study investigates the interaction between elevated BNP and QTc prolongation on short-term mortality in SHS patients. This cohort study included patients with suspected AIS and concomitant SHS. SHS is defined as new-onset cardiac dysfunction after AIS, including acute coronary syndrome, heart failure, and arrhythmias. All patients underwent laboratory tests and electrocardiographic evaluations. Prolonged QTc was defined as >430 milliseconds (ms) in males and >450 ms in females. Patients were followed up for three months, and the study outcomes were all-cause mortality and cardiovascular and cerebrovascular disease (CCVD) mortality. Cox regression models were used to assess the relationship between BNP, QTc interval, and mortality, and the interaction between BNP and the QTc interval on mortality was analyzed. A total of 448 patients were enrolled in this analysis. Prolonged QTc was present in 217 patients (48.44%). Elevated BNP was associated with prolonged QTc (OR 1.90; 95% CI, 1.20-3.01, p=0.006). Elevated BNP, but not prolonged QTc, increased the risk of all-cause and CCVD mortality (HR 5.94; 95% CI, 1.22-29.03, p=0.028 and HR 5.48; 95% CI, 1.01-29.70, p=0.048, respectively). There was a significant interaction between elevated BNP and prolonged QTc on all-cause mortality (p for interaction <0.001). Patients with prolonged QTc and higher BNP had highest risk of all-cause mortality (HR 4.92, 95% CI, 1.03-23.39, p=0.045). This study highlights a significant interaction between prolonged QTc and elevated BNP levels in predicting short-term all-cause mortality among AIS patients with SHS. Prolonged QTc emerges as a critical marker of increased mortality risk, particularly in patients with elevated BNP levels.

## INTRODUCTION

Acute ischemic stroke (AIS) is a prevalent cerebrovascular disease associated with significant risk of disability and mortality [1, 2]. Beyond its neurological effects, AIS induces complex alterations in cardiac function, highlighting the interconnected nature of the brain and heart [3-5]. Studies estimate that approximately 19% of AIS patients experience at least one severe cardiac adverse event shortly after disease onset [6]. To capture these interactions, the concept of stroke-heart syndrome has emerged, describing the deterioration of cardiac function or changes in the heart following ischemic stroke [7].

The intricate physiological and neurohumoral networks of the brain-heart axis are central to post-stroke cardiac complications [8, 9]. Key mechanisms include activation of the hypothalamic-pituitary-adrenal axis, disruption of the autonomic nervous system, and inflammatory and immune responses [10, 11]. These processes disrupt normal cardiac rhythm, leading to abnormal electrocardiographic findings [12]. Among these, prolonged QTc interval is the most common electrocardiographic abnormality observed in patients with stroke-heart syndrome [13]. QTc prolongation reflects disturbances in cardiac repolarization, which may signal an increased risk of arrhythmia and sudden death in stroke patients [14]. Similarly, elevated brain natriuretic peptide (BNP) levels are often associated with cardiac insufficiency and greater stroke severity [15]. Despite this, the interplay between BNP elevation and QT prolongation, and their impact on short-term mortality risk, remains poorly understood.

In emergency settings, rapid and accurate assessment of short-term mortality risk in AIS patients with cardiac complications is critical for optimizing clinical decision-making [16, 17]. While prior research has explored the predictive role of BNP and QTc prolongation in ischemic stroke, their interaction within the context of stroke-heart syndrome has yet to be fully characterized. This study aims to investigate the combined impact of QTc prolongation and elevated BNP levels on short-term mortality risk in stroke-heart syndrome patients.

## MATERIALS AND METHODS

### Data source and study setting

This retrospective cohort study was conducted using data from the electronic medical records system in Xuanwu Hospital, Capital Medical University. The system provides comprehensive patient information, including demographic details, medical history, laboratory results, imaging reports, and clinical notes. The study included patients who presented to the Emergency Department of Xuanwu Hospital, Capital Medical University, between January 2021 and July 2022, with suspected AIS and concomitant SHS. The study was conducted in accordance with the principles of the Declaration of Helsinki. The study included patients who presented with focal neurological symptoms related to AIS (confirmed by computed tomography [CT] or magnetic resonance imaging [MRI] of the head) and new-onset cardiac dysfunction within three days of stroke onset. Baseline data, clinical characteristics, vital signs, electro-cardiogram (ECG) parameters, and laboratory findings were retrospectively collected. Patients were divided into two groups based on the presence or absence of a prolonged QTc interval on ECG on admission. A three-month telephone follow-up was conducted to obtain survival information. The study was approved by the Ethics Committee of Xuanwu Hospital of Capital Medical University (Approval number: [2019] 012). Informed consent was waived due to the nature of the study design.

### Inclusion and Exclusion Criteria

Inclusion criteria were as follows: (1) patients with acute focal neurological deficits confirmed by imaging as AIS; (2) age greater than 18 years; (3) presence of SHS, defined as new-onset cardiac dysfunction within three days of AIS onset, meeting the World Stroke Organization Brain & Heart Task Force criteria for stroke-heart syndrome, defined by at least one of the following: (a) acute myocardial injury, either ischemic or non-ischemic, characterized by elevated cardiac troponin levels; (b) acute myocardial infarction following a stroke; (c) left ventricular dysfunction, heart failure, or post-stroke Takotsubo cardiomyopathy; (d) electrocardiogram abnormalities and cardiac arrhythmias, including post-stroke atrial fibrillation; (e) sudden cardiac death of neurogenic origin after stroke[18].

Exclusion criteria: (1) pre-stroke modified Rankin Scale (mRS) > 2; (2) coexisting conditions on admission that cause elevated cTn levels, such as sepsis, acute kidney injury, major cardiac surgery, deep vein thrombosis, pulmonary embolism, or pre-existing cardiac dysfunction (e.g., myocardial infarction, congestive heart failure) within two weeks prior; (3) type-1 myocardial infarction; (4) patients with missing BNP or ECG measurement data; (5) participation in other clinical trials.

### Data Collection

Demographic information included age and sex. Clinical parameters collected included the National Early Warning Score (NEWS), diastolic blood pressure (DBP), systolic blood pressure (SBP), temperature, oxygen saturation (SaO_2_), respiratory rate (RR), and heart rate (HR). Routine laboratory tests were conducted for all patients, and findings included BNP, high sensitivity cardiac troponin I (hs-cTnI), blood urea nitrogen (BUN), creatinine (Crea), uric acid (UA), albumin (Alb), creatine kinase (CK), glucose (Glu), serum calcium, serum potassium, white blood cell count (WBC), neutrophil count (NEUT), and D-dimer levels. For all included patients, blood samples for BNP measurement were obtained upon admission to the emergency department prior to any major intervention or treatment, within three days of AIS symptom onset. All data were obtained directly from the electronic medical record system by trained investigators.

### QTc Measurement

All patients underwent a single 12-lead ECG upon admission to the emergency department within three days of stroke onset. The ECG tracings were recorded on paper strips at a speed of 25 mm/s and an amplitude of 10 mm/mV. The QT interval was defined as the time from the onset of the Q wave to the end of the T wave, representing the total duration of ventricular depolarization and repolarization. To measure the QT interval, three to five consecutive waves were recorded, and the average was calculated. Lead II was selected as the most appropriate reference lead. QTc interval was correction for heart rate using Bazett's formula. Based on previous literature, QTc prolongation was defined as a QTc interval greater than 430 ms in males and 450 ms in females [19].

### Outcomes

The primary outcome was all-cause mortality within 90 days of symptom onset, and the secondary outcome was cardiovascular and cerebrovascular disease (CCVD) mortality. CCVD mortality was defined as deaths directly attributable to cardiovascular or cerebrovascular events. These events included myocardial infarction, heart failure, fatal arrhythmias such as ventricular fibrillation, cardiac arrest, and major vascular ruptures such as aortic dissection. Additionally, cerebrovascular events such as stroke (both ischemic and hemorrhagic), ruptured cerebral aneurysms, and intracerebral hemorrhage were also considered. Mortality status and causes were collected by reviewing readmission records or contacting the relatives by telephone. The researchers used a standardized, structured questionnaire to ensure the accuracy of the interviews.

**Table 1:** Baseline characteristics between patients with or without QTc prolongation.

	Total (n = 448)	QTc normal (n = 231)	QTc prolonged (n = 217)	p-value
**Age, year**	67.2 ± 12.5	68.2 ± 12.1	66.2 ± 12.8	0.091
**Gender, n (%)**				0.007
**Female**	143 (31.9)	87 (37.7)	56 (25.8)	
**Male**	305 (68.1)	144 (62.3)	161 (74.2)	
**NEWS**	1.1 ± 1.1	1.0 ± 1.1	1.2 ± 1.1	0.072
**DBP, mmHg**	80.7 ± 22.5	80.6 ± 20.9	80.8 ± 24.1	0.902
**SBP, mmHg**	153.4 ± 42.6	156.9 ± 40.1	149.7 ± 44.8	0.073
**Temperature, °C**	34.4 ± 7.8	34.4 ± 7.7	34.3 ± 8.0	0.892
**SaO_2_, %**	92.4 ± 20.9	93.1 ± 19.5	91.7 ± 22.4	0.474
**RR, times/min**	18.7 ± 4.3	18.8 ± 3.9	18.6 ± 4.6	0.52
**HR, beats/min**	74.6 ± 14.3	69.8 ± 12.1	79.6 ± 14.7	< 0.001
**BNP, pg/mL**	932.2 ± 3485.1	591.2 ± 2668.4	1295.3 ± 4159.0	0.032
**hs-cTnI, ng/mL**	0.0 ± 0.4	0.0 ± 0.0	0.1 ± 0.5	0.053
**BUN, mmol/L**	7.0 ± 4.6	6.7 ± 3.3	7.4 ± 5.6	0.143
**Crea, μmoI/L**	83.0 ± 83.7	78.8 ± 65.7	87.5 ± 99.2	0.273
**UA, μmol/L**	340.3 ± 113.3	335.3 ± 99.4	345.5 ± 126.4	0.344
**Alb, g/L**	40.9 ± 4.8	41.4 ± 4.5	40.2 ± 4.9	0.008
**CK, U/L**	156.7 ± 537.8	119.4 ± 196.0	195.9 ± 743.0	0.144
**Glu, mmol/L**	9.3 ± 5.1	9.0 ± 4.5	9.6 ± 5.7	0.231
**Serum calcium, mmol/L**	2.3 ± 0.2	2.4 ± 0.2	2.3 ± 0.2	0.06
**Serum potassium, mmol/L**	4.2 ± 0.5	4.2 ± 0.5	4.1 ± 0.5	0.004
**WBC, 10^9**	8.0 ± 9.7	7.1 ± 2.5	8.9 ± 13.7	0.057
**NEUT, 10^9**	5.6 ± 4.9	5.1 ± 2.4	6.2 ± 6.6	0.019
**D-dimer, Mean ± SD, mg/L**	7.1 ± 4.0	6.8 ± 4.0	7.4 ± 3.9	0.181

Abbreviations: BNP, brain natriuretic peptide; QTc, corrected QT interval; NEWS, National Early Warning Score; DBP, diastolic blood pressure; SBP, systolic blood pressure; SaO_2_, oxygen saturation; RR, respiratory rate; HR, heart rate; hs-cTnI, high sensitivity cardiac troponin I; BUN, blood urea nitrogen; Crea, creatinine; UA, uric acid; Alb, albumin; CK, creatine kinase; Glu, glucose; WBC, white blood cell; NEUT, neutrophil

### Statistical analysis

All normally distributed continuous variables were presented as mean ± SD. Categorical variables were presented as frequencies (%). Comparisons of continuous variables among groups were performed with the use of the independent samples Student’s t-test or Mann-Whitney U-test depending on the normality of the distribution, and categorical data were compared by chi-square test, as appropriate. The optimal cutoff value of BNP for predicting mortality outcomes was determined using a Receiver Operating Characteristic (ROC) curve. The effect of BNP on QTc intervals was evaluated using linear regression models (regression coefficients β and 95% CI) with adjustment for major covariates. Logistic regression was applied to investigate the associations between BNP and QTc prolongation. Model I was adjusted for age and sex. Model II was additionally adjusted for NEWS, SBP, serum potassium and neutrophil count. Since the National Institutes of Health Stroke Scale (NIHSS) was not available in our dataset, the NEWS score was included in the adjusted models as a limited surrogate marker of physiological instability, rather than as a proxy for stroke severity. Therefore, the potential confounding effects of stroke severity could not be fully accounted for in our analysis. We conducted restricted cubic spline (RCS) model to develop smooth curves to examine the possible nonlinear dose-response associations between BNP and QTc prolongation. In this model, BNP was used as a continuous variable with four knots (5th, 35th, 65th and 95th). Nonlinearity was assessed using a likelihood ratio test, comparing a model with only a linear term to a model incorporating both linear and cubic spline terms. Multivariate cox regression models were performed to calculate hazard ratios (HR) and 95% CI for all-cause and CCVD mortality. Model I was adjusted for age and sex, and model II was additionally adjusted for NEWS, SBP and neutrophil count. Confounders were selected based on clinical interest, previous scientific literatures, and significant covariates in the univariate analysis. Kaplan-Meier survival curves were generated to depict mortality status in patients with and without elevated BNP or QTc prolongation, with comparisons evaluated using the log-rank test. The interaction effect between BNP and QTc prolongation on mortality outcomes was assessed in the regression models. Statistical significance was defined as a two-sided p-value < 0.05. Missing data were processed using multiple imputations. All analyses were conducted using R version 4.2.1 (www.R-project.org), developed by the R Foundation.

**Table 2:** The association between BNP and QTc interval in patients with stroke-heart syndrome.

	Crude		Model I		Model II	
Linear regression^*^	**Coefficient (95%CI)**	**p value**	**Coefficient (95%CI)**	**p value**	**Coefficient (95%CI)**	**p value**
**BNP per 100 pg/mL**	0.14 (0.06~0.22)	0.001	0.13 (0.05~0.21)	0.002	0.13 (0.04~0.21)	0.005
**BNP<100 pg/mL**	**Ref**		**Ref**		**Ref**	
**BNP≥100 pg/mL**	13.93 (8.38~19.48)	<0.001	13.55 (7.48~19.62)	<0.001	11.34 (5.12~17.55)	<0.001
**BNP<122.5 pg/mL**	**Ref**		**Ref**		**Ref**	
**BNP≥122.5 pg/mL**	14.15 (8.64~19.65)	<0.001	13.73 (7.81~19.65)	<0.001	12.45 (6.32~18.59)	<0.001
**Logistic regression^#^**	**OR (95%CI)**	**p value**	**OR (95%CI)**	**p value**	**OR (95%CI)**	**p value**
**BNP per 100 pg/mL**	1.01 (1~1.01)	0.052	1.01 (1~1.02)	0.023	1.01 (1~1.02)	0.018
**BNP<100 pg/mL**	**Ref**		**Ref**		**Ref**	
**BNP≥100 pg/mL**	1.26 (0.87~1.84)	0.219	1.71 (1.12~2.62)	0.013	1.48 (0.94~2.33)	0.092
**BNP<122.5 pg/mL**	**Ref**		**Ref**		**Ref**	
**BNP≥122.5 pg/mL**	1.5 (1.04~2.18)	0.032	2.05 (1.34~3.12)	0.001	1.9 (1.2~3.01)	0.006

BNP, brain natriuretic peptide; QTc, corrected QT interval. Model I was adjusted for age and sex. Model II was additionally adjusted for NEWS, SBP, serum potassium and neutrophil count. *Linear regression analyses assessed the association between BNP levels and continuous changes in the QTc interval; #Logistic regression analyses assessed the relationship between BNP levels and QTc prolongation.

## RESULTS

### Baseline Characteristics

The study included 448 patients who met the inclusion criteria (see Supplementary Fig. 1 for the flowchart). All patients were followed up for 3 months. Of these, 104 patients were excluded due to missing BNP data, and 12 patients were lost to follow-up. During follow-up, there were 14 deaths, of which 9 (64.3%) were CCVD death. The mean age of the total study population was 67.2 years, and 68.1% of the patients were male. The patients were divided into two groups based on whether their QTc interval was prolonged. Compared to the normal QTc group, the prolonged QTc group had a higher proportion of male patients and faster baseline heart rates. BNP levels, Alb, serum potassium, and neutrophil count were significantly higher in the prolonged QTc group. No significant differences were found in other characteristics between the two groups (Table 1).

**Figure 1.**
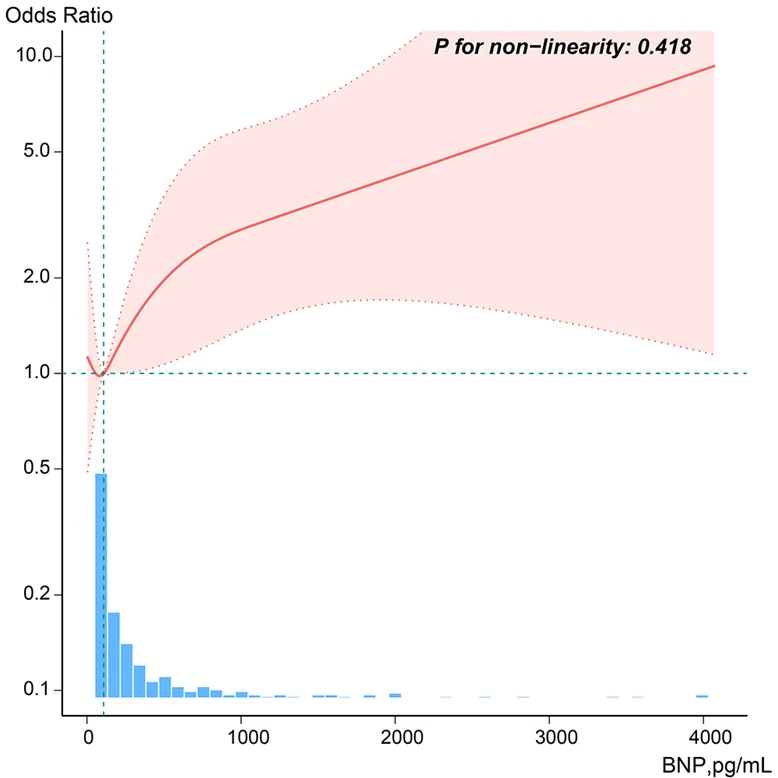
**RCS plot depicting the association between BNP levels and the risk of QTc prolongation**. As BNP level increases, the risk of QTc prolongation rises proportionally in patients with SHS. The solid line represents the estimated odds ratio for QTc prolongation across the range of BNP values, while the shaded area indicates the 95% confidence interval.

### Regression Analysis Between BNP and QTc

The first step of the study explored the relationship between BNP levels and QTc prolongation in the SHS population (Table 2). Based on previous literature, patients were divided into two groups: BNP ≥100 pg/mL and BNP <100 pg/mL. Additionally, an ROC curve analysis determined the optimal BNP cutoff value to be 122.5 pg/mL (area under the ROC curve 0.735, sensitivity 85.7%, specificity 46.5%) (Supplementary Fig. 2). This cutoff effectively distinguished patients at higher risk of mortality. A linear regression analysis assessed the association between BNP levels and continuous changes in the QTc interval. For every 100 pg/mL increase in BNP, the QTc interval increased by an average of 0.14 ms (95% CI 0.06-0.22, p = 0.001). After adjusting for potential confounders, the association remained significant (Model I: 0.13, 95% CI 0.05-0.21, p = 0.002; Model II: 0.13, 95% CI 0.04-0.21, p = 0.005). When BNP was ≥100 pg/mL or 122.5 pg/mL, the QTc interval increased by 11.34 ms and 12.45 ms, respectively. A logistic regression analysis was used to assess the relationship between BNP levels and QTc prolongation. Compared to patients with BNP <122.5 pg/mL, patients with BNP ≥122.5 pg/mL have a higher risk of QTc prolongation (OR 1.5, 95% CI [1.04-2.18], p = 0.032), and the results were robust after adjusting for confounders (OR 1.9, 95% CI [1.2-3.01], p = 0.006).

An RCS curve was used to explore the dose-response relationship between BNP levels and the risk of QTc prolongation (Fig. 1). The results demonstrated a significant linear dose-response relationship between BNP levels and the risk of QTc prolongation. As BNP levels increased, the risk of QTc prolongation rose proportionally. The reference BNP value in the RCS analysis was the median BNP of 108.5 pg/mL, indicating a lower risk of QTc prolongation below this level. The p-value for non-linearity was 0.418, indicating that the relationship between BNP and QTc prolongation can be considered linear.

**Figure 2.**
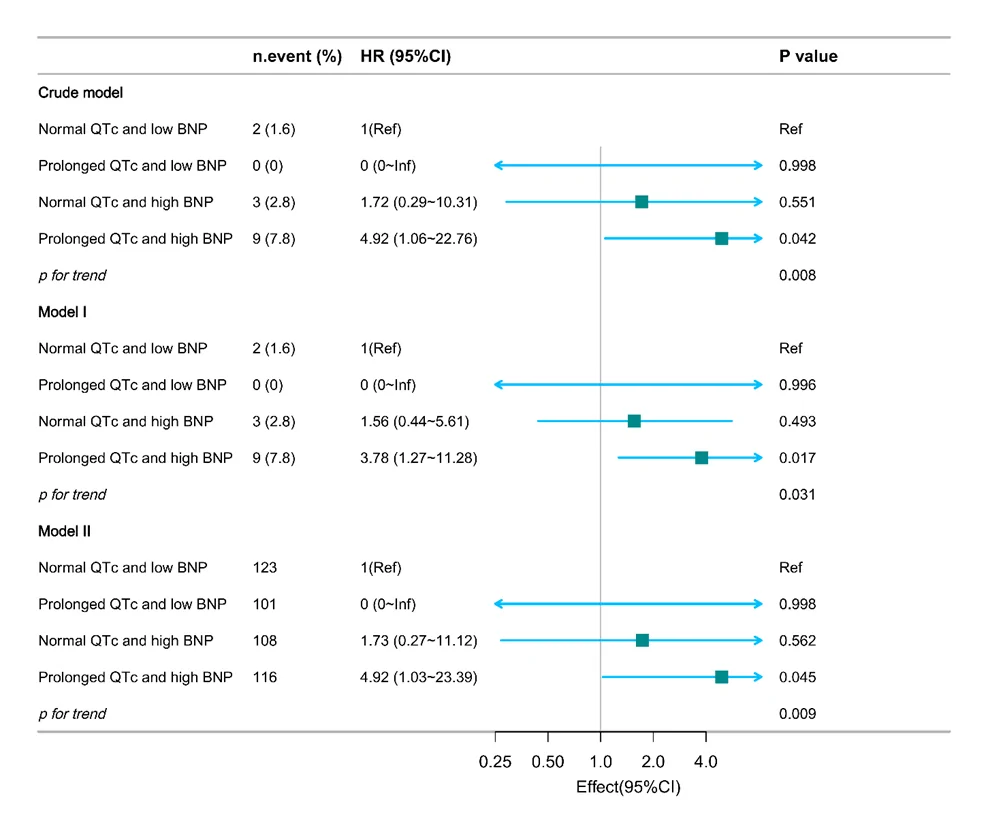
**Synergistic effect of BNP and QTc prolongation on all-cause mortality**. Patients were categorized into four groups based on QTc interval status (normal vs. prolonged) and BNP level (low <122.5 pg/mL vs. high ≥122.5 pg/mL). Model I was adjusted for age and sex, and model II was additionally adjusted for NEWS, SBP and neutrophil count. Patients with both prolonged QTc and high BNP exhibited the greatest risk of all-cause mortality.

### Correlation Between QTc, BNP, and mortality

During the 3-month follow-up period, 14 patients died, of whom 9 (64.29%) died due to CCVD. Univariate Cox regression analysis identified potential risk factors for all-cause mortality within 3 months in patients with stroke-heart syndrome (Supplementary Table 1). The following were identified as potential risk factors for all-cause mortality: National Early Warning Score (NEWS), elevated BNP levels, decreased serum albumin, elevated creatine kinase, elevated glucose, decreased serum calcium, and increased heart rate. Elevated creatine kinase, decreased serum calcium, increased heart rate, QTc prolongation, and elevated BNP levels were potential risk factors for CCVD mortality. For every 10 ms increase in the QTc interval, the risk of all-cause mortality increased by 18%. However, QTc prolongation was not significantly associated with all-cause mortality. BNP levels, both for every 100 pg/mL increase and at levels of 100 pg/mL and 122.5 pg/mL, were significantly associated with an increased risk of all-cause mortality.

After adjusting for covariates, we evaluated the impact of BNP levels and QTc intervals on 3-month all-cause mortality and CCVD mortality (Table 3). The results showed that for every 100 pg/mL increase in BNP, there was a slight increase in the risk of all-cause mortality (Model II HR 1.01, 95% CI 1.00-1.02, p = 0.009). For every 10 ms increase in the QTc interval, there was a significant association with increased all-cause mortality in the unadjusted model (Crude HR 1.18, 95% CI 1.06-1.32, p = 0.003), but after adjusting for confounders, the statistical significance weakened (Model II HR 1.15, 95% CI 0.99-1.32, p = 0.06). A 10 ms increase in the QTc interval was significantly associated with CCVD mortality (Model II HR 1.20, 95% CI 1.00-1.45, p = 0.046). BNP ≥122.5 pg/mL was significantly associated with higher risk of all-cause and CCVD mortality (Model II HR 5.94, 95% CI 1.22-29.03, p = 0.028; HR 5.48, 95% CI 1.01-29.70, p = 0.048). Notably, QTc prolongation did not show a significant association with all-cause and CCVD mortality (Model II HR 1.69, 95% CI 0.52-5.48, p = 0.379; HR 1.18, 95% CI 0.3-4.57, p = 0.816).

**Table 3:** Multivariate cox regression analysis of BNP, QTc intervals, QTc prolongation, and all-cause and CCVD mortality.

	N. total	N. event (%)	Crude HR (95% CI)	p value	Model I HR (95% CI)	p value	Model II HR (95%CI)	p value
**All-cause mortality**								
**BNP per 100 pg/mL**			1.01 (1.00,1.01)	0.010	1.01 (1.00,1.01)	0.009	1.01 (1.00,1.02)	0.009
**QTc per 10ms**			1.18 (1.06,1.32)	0.003	1.18 (1.05,1.32)	0.006	1.15 (0.99~1.32)	0.060
**BNP<100 pg/mL**	195	2 (1)	1 (Ref)		1(Ref)		1(Ref)	
**BNP>=100 pg/mL**	253	12 (4.7)	4.71 (1.05~21.06)	0.040	4.9 (1.02~23.47)	0.046	4.14 (0.84~20.44)	0.081
**BNP<122.5 pg/mL**	234	2 (0.9)	1 (Ref)		1 (Ref)		1(Ref)	
**BNP>=122.5 pg/mL**	214	12 (5.6)	6.72 (1.5~30.02)	0.010	7.22 (1.54~34)	0.012	5.94 (1.22~29.03)	0.028
**Normal QTc**	231	5 (2.2)	1 (Ref)		1(Ref)		1(Ref)	
**Prolonged QTc**	217	9 (4.1)	1.93 (0.65~5.77)	0.240	2.05 (0.68~6.17)	0.204	1.69 (0.52~5.48)	0.379
**CCVD mortality**								
**BNP per 100 pg/mL**			1.01 (1.00~1.02)	0.290	1.01 (1.00~1.02)	0.246	1.01 (1.00~1.02)	0.158
**QTc per 10ms**			1.16 (1~1.35)	0.050	1.18 (1.01~1.38)	0.038	1.2 (1~1.45)	0.046
**BNP<100 pg/mL**	195	2 (1)	1 (Ref)		1 (Ref)		1(Ref)	
**BNP>=100 pg/mL**	253	7 (2.8)	2.73 (0.57~13.14)	0.210	3.36 (0.64~17.58)	0.15	3.62 (0.66~19.91)	0.139
**BNP<122.5 pg/mL**	234	2 (0.9)	1 (Ref)		1 (Ref)		1(Ref)	
**BNP>=122.5 pg/mL**	214	7 (3.3)	3.88 (0.81~18.7)	0.091	4.85 (0.95~24.87)	0.058	5.48 (1.01~29.7)	0.048
**Normal QTc**	231	4 (1.7)	1(Ref)		1(Ref)		1(Ref)	
**Prolonged QTc**	217	5 (2.3)	1.33 (0.36~4.97)	0.668	1.28 (0.34~4.82)	0.713	1.18 (0.3~4.57)	0.816

BNP, brain natriuretic peptide; QTc, corrected QT interval. CCVD, cardiovascular and cerebrovascular disease. Model I was adjusted for age and sex, and model II was additionally adjusted for NEWS, SBP and neutrophil count.

We further analyzed the interaction between BNP levels and QTc interval on 3-month all-cause and CCVD mortality (Table 4 and Supplementary Table 2). When BNP levels were below 100 pg/mL, no significant association was found between a 10 ms increase in QTc and increased mortality risk (Model II HR 0.78, p = 0.52). However, when BNP levels were ≥100 pg/mL, a potential association emerged (Model II HR 1.13, p = 0.083). Similarly, when BNP levels were below 122.5 pg/mL, no significant association was found between QTc and mortality risk (Model II HR 0.77, p = 0.513). When BNP levels exceeded 122.5 pg/mL, a potential association was observed (Model II HR 1.12, p = 0.125). However, no significant interaction between QTc increase and BNP levels on mortality outcomes was detected. When BNP <100 pg/mL or <122.5 pg/mL, QTc prolongation was not associated with short-term mortality. When BNP levels were ≥100 pg/mL or ≥122.5 pg/mL, the association between QTc prolongation and short-term mortality risk approached significance. The interaction between QTc prolongation and BNP levels (<100 pg/mL or <122.5 pg/mL) for all-cause mortality was significant (p < 0.001; p < 0.001), suggesting a synergistic effect of BNP levels and QTc prolongation on mortality risk. For CCVD mortality, the interaction between QTc prolongation and BNP levels approached near significance (p = 0.063; p = 0.078).

**Figure 3.**
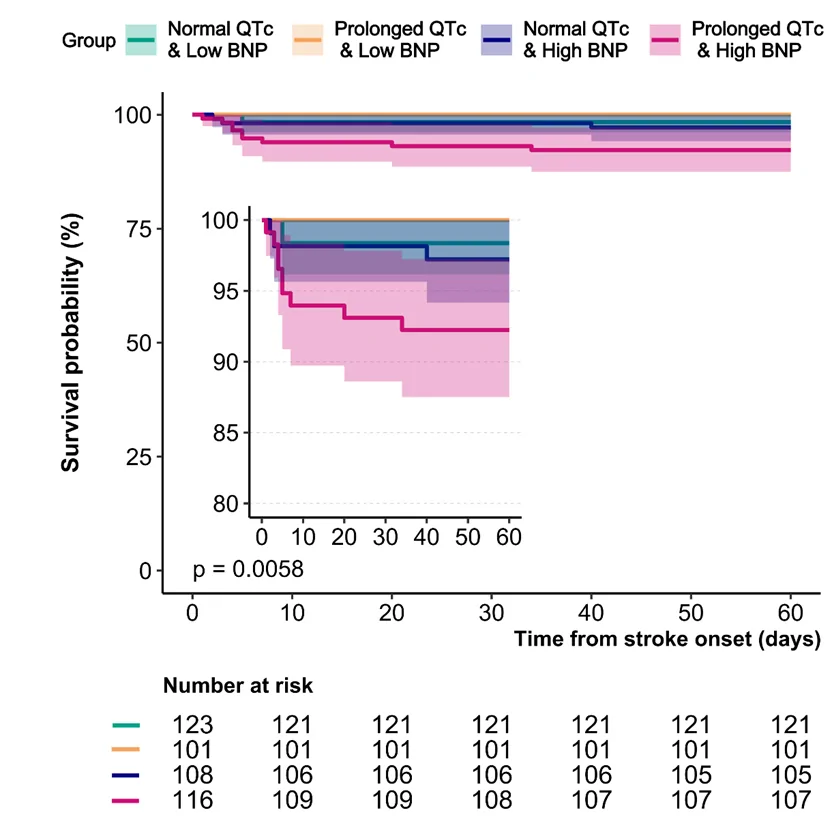
**Kaplan-Meier survival curves for all-cause mortality within 90 days after stroke onset, stratified by QTc interval and BNP level**. Patients were divided into four groups based on the presence or absence of QTc prolongation and BNP elevation (BNP ≥122.5 pg/mL): normal QTc and low BNP; prolonged QTc and low BNP; normal QTc and high BNP; prolonged QTc and high BNP. The x-axis represents time from stroke onset (days), and the y-axis represents cumulative survival probability. Patients with prolonged QTc and high BNP exhibited the lowest survival probability, with the majority of deaths occurring within the first two weeks after stroke onset.

Patients were further divided into four groups based on BNP elevation (BNP ≥122.5 pg/mL) and QTc prolongation (normal QTc and low BNP; prolonged QTc and low BNP; normal QTc and high BNP; prolonged QTc and high BNP). Multivariate Cox regression analysis showed that, after full adjustment, compared to the reference group (normal QTc and low BNP), patients with prolonged QTc and high BNP had a significantly higher risk of mortality (HR 4.92, 95% CI 1.03-23.39, p = 0.045) (Fig. 2). The Kaplan-Meier survival curves displayed the 3-month survival probabilities for the four groups (Fig. 3). Patients with both prolonged QTc and elevated BNP had the highest risk of all-cause mortality within 3 months, with the majority of deaths occurring within the first two weeks after stroke onset.

## DISCUSSION

The QTc interval, a key ECG indicator, represents the time from ventricular depolarization to complete repolarization. Prolongation of QTc interval is associated with malignant ventricular arrhythmias [20]. Studies have shown that QTc prolongation is an important predictor of both short-term and long-term adverse outcomes in patients with cardiovascular diseases [21]. However, in acute ischemic stroke (AIS) patients, the role of the QTc interval in risk stratification for cardiac complications remains inadequately supported by clinical evidence. Patients who develop cardiovascular complications post-stroke experience significantly higher all-cause mortality and readmission rates within five years compared to those without such complications [22]. The emerging stroke-heart syndrome concept has shifted research focus toward understanding its risk factors, including insular infarction and vascular comorbidities [23, 24]. Despite this progress, prognostic studies and clinical guidelines addressing this population remain limited. Given the short disease course and poor patient outcomes, it is essential to explore convenient and sensitive indicators for early prognosis management. To address this gap, we hypothesized that QTc interval monitoring, particularly stratified by BNP levels, could identify high-risk stroke-heart syndrome patients.

**Table 4:** The interaction analyses between BNP and QTc on all-cause mortality.

BNP groups	QTc intervals	N.total	N.event (%)	Crude HR (95%CI)	p value	Model I HR (95%CI)	p value	Model II HR (95%CI)	p value	p for interaction
**BNP<100 pg/mL**	QTc per 10ms	195	2 (1)	0.79 (0.43~1.44)	0.44	0.81 (0.44~1.48)	0.493	0.78 (0.37~1.65)	0.52	0.227
**BNP>=100 pg/mL**	QTc per 10ms	253	12 (4.7)	1.17 (1.04~1.31)	0.008	1.17 (1.04~1.31)	0.009	1.13 (0.98~1.31)	0.083	
**BNP<122.5 pg/mL**	QTc per 10ms	234	2 (0.9)	0.78 (0.43~1.42)	0.418	0.81 (0.44~1.48)	0.486	0.77 (0.36~1.67)	0.513	0.214
**BNP>=122.5 pg/mL**	QTc per 10ms	214	12 (5.6)	1.15 (1.03~1.29)	0.015	1.16 (1.03~1.3)	0.015	1.12 (0.97~1.29)	0.125	
BNP<100 pg/mL	Normal QTc	107	2 (1.9)	1(Ref)		1(Ref)		1(Ref)		<0.001
	Prolonged QTc	88	0 (0)	0 (0~Inf)	0.999	0 (0~Inf)	0.999	0 (0~Inf)	0.927	
BNP>=100 pg/mL	Normal QTc	124	3 (2.4)	1(Ref)		1(Ref)		1(Ref)		
	Prolonged QTc	129	9 (7)	2.94 (0.8~10.86)	0.106	3.02 (0.8~11.35)	0.102	2.48 (0.61~10.13)	0.206	
BNP<122.5 pg/mL	Normal QTc	132	2 (1.5)	1(Ref)		1(Ref)		1(Ref)		<0.001
	Prolonged QTc	102	0 (0)	0 (0~Inf)	0.999	0 (0~Inf)	0.999	0 (0~Inf)	0.997	
BNP>=122.5 pg/mL	Normal QTc	99	3 (3)	1(Ref)		1(Ref)		1(Ref)		
	Prolonged QTc	115	9 (7.8)	2.63 (0.71~9.73)	0.146	2.71 (0.71~10.33)	0.144	2.22 (0.53~9.25)	0.273	

BNP, brain natriuretic peptide; QTc, corrected QT interval. Model I was adjusted for age and sex, and model II was additionally adjusted for NEWS, SBP and neutrophil count.

Our study revealed a significant correlation between elevated BNP levels and prolonged QTc intervals. BNP may modulate ion channel function and action potential duration by inhibiting sympathetic nervous system excitation and reducing catecholamine release [25]. Prolonged action potential duration is typically associated with QT prolongation [26]. For example, BNP has been shown to prolong action potential duration and reduce transient outward potassium currents in rat hearts, an adaptation that aims to maintain contractility in a failing heart but also increases the risk of arrhythmias [25]. The prolongation of the QTc interval may partially mediate the adverse prognostic impact of elevated BNP. However, the exact biological mechanism underlying the relationship between BNP and QTc prolongation remains unclear and warrants further research to better understand the factors influencing prognosis in stroke-heart syndrome patients.

Additionally, our study found that elevated BNP levels significantly increased the short-term mortality risk in stroke-heart syndrome patients. In stroke-heart syndrome patients, BNP levels significantly impact on the short-term prognosis of these patients, as they indicate excessive sympathetic activation and potential cardiac dysfunction. Stroke can damage the Central Autonomic Network (CAN), leading to excessive sympathetic activation and over-secretion of catecholamines [27, 28]. This exacerbates post-stroke inflammation, resulting in cardiac injury and increased BNP secretion [29, 30]. Numerous studies have confirmed that elevated BNP levels after stroke are associated with poor outcomes, particularly in the context of heart failure, where elevated BNP is significantly linked to short-term mortality [31, 32].

However, we observed that QTc prolongation did not significantly increase short-term mortality risk in the entire patient population. Our findings are consistent with those of Henninger et al., who reported that QTc prolongation did not significantly increase adverse outcomes within three months in AIS patients [33]. Hromádka et al. found that QTc prolongation after 48 hours in AIS patients was associated with one-year mortality [12]. Numerous cohort studies have also demonstrated that QTc prolongation increases long-term all-cause mortality and cardiovascular mortality in healthy populations [34, 35]. This suggests that QTc prolongation may have a greater impact on the long-term prognosis of stroke patients. Furthermore, we found that the effect of QTc prolongation on short-term mortality was more pronounced in patients with elevated BNP levels, whereas in patients without elevated BNP, the effect of QTc prolongation on mortality risk was smaller or negligible. This interaction suggests that the predictive value of QTc prolongation for short-term mortality depends on the patient's cardiac function, particularly BNP, a key marker of cardiac function. The reason for this difference may be that patients with severe cardiac function are more prone to increased QT dispersion (QTd) and abnormal excitation-contraction coupling, both of which are important mechanisms through which QTc prolongation leads to sudden cardiac death [36, 37]. Alicia et al. also confirmed that in healthy individuals without cardiovascular disease, QTc prolongation was not strongly associated with cardiovascular events and death, whereas in patients with diagnosed cardiovascular disease, it was associated with increased overall mortality, cardiovascular mortality, and sudden death risk [38]. High BNP levels indicate more severe cardiac dysfunction and cardiovascular disease after stroke, making patients more susceptible to these mechanisms of sudden cardiac death. Furthermore, post-stroke autonomic dysfunction may increase QTd and exacerbate abnormal excitation-contraction coupling, interacting with other factors that mediate cardiovascular events and sudden death [39]. Therefore, further research into the mechanisms of interaction between BNP and QTc intervals will help improve clinical management and risk assessment of stroke-heart syndrome patients.

In this study, we found a notable interaction between prolonged QTc intervals and elevated BNP levels in predicting short-term all-cause mortality in stroke-heart syndrome patients. This aligns with previous findings. In heart failure patients with BNP levels >400 pg/mL, QTc prolongation is a strong and independent predictor of adverse outcomes [40]. Lin et al. reported that myocardial infarction patients with both elevated BNP and prolonged QTc had significantly higher short-term risks of all-cause mortality and readmission compared to those with either elevated BNP or prolonged QTc alone [41]. Similarly, Vrtovec et al. found that elevated BNP in heart failure patients was associated with an increased risk of cardiac death only in patients with prolonged QTc [42]. The simultaneous occurrence of elevated BNP and prolonged QTc may indicate that the heart is not only under significant mechanical stress but also experiencing electrophysiological instability. This dual stress increases the risk of malignant arrhythmias and cardiac dysfunction, leading to a higher likelihood of short-term all-cause mortality and CCVD-related death. Our results showed that the interaction between QTc prolongation and BNP levels on CCVD mortality was nearly significant, possibly due to insufficient statistical power caused by the small sample size. From a basic research perspective, the interaction between BNP and the QTc interval may result from the combined effects of electrophysiological regulation at the myocardial cell level and systemic neurohumoral regulation [43]. Clinically, our findings suggest that QTc interval monitoring should be prioritized in stroke-heart syndrome patients with elevated BNP levels to identify potential arrhythmia risks early and adjust treatment strategies to reduce mortality. These insights are critical for the individualized management of stroke-heart syndrome patients.

The mortality rate reported in our study was lower than that reported in previous literature, which may be due to the fact that our study mainly analyzed patients with SHS within 3 days after stroke, which may underestimate the overall mortality of SHS [44]. Recent large-scale evidence indicates that while the incidence of SHS peaks within the first 3 days after stroke, patients developing SHS between 10- and 30-days post-stroke have a significantly higher risk of death compared with those with early-onset SHS [45]. This suggests that late-onset SHS may present with a distinct and more severe risk profile, underscoring the importance of monitoring for cardiac complications throughout the subacute phase after stroke. It is also worth noting that the cases in this study come from a tertiary general hospital with strong acute management and comprehensive intervention capabilities, which help reduce short-term mortality; in addition, the follow-up time is relatively short (3 months), which may underestimate the long-term risk of death.

Our study has several limitations. As a single-center retrospective study conducted at a tertiary care hospital, our findings may be influenced by referral bias, potentially limiting the generalizability of our results to other settings or populations. The BNP and QTc levels are only measured once, and future prospective studies should incorporate serial BNP and QTc monitoring to better understand the prognostic value of dynamic changes in BNP in this patient population. The BNP cutoff in this study was internally derived from our SHS population and may not be generalizable to other cohorts. Therefore, this threshold requires external validation in larger, multicenter studies before it can be widely adopted in clinical practice. Another important consideration is that our analysis did not adjust for certain neurological variables, such as stroke severity scores (e.g., NIHSS) and infarct location. Both stroke severity and infarct location may independently influence cardiac function. The absence of these key variables in our regression models introduces the possibility of residual confounding. Additionally, the total number of mortality events in our cohort was relatively low (n=14), which may increase the risk of model overfitting. As a result, the findings regarding independent predictors of mortality and the interaction between BNP and QTc should be interpreted with caution, and validation in larger cohorts is warranted.

This single-center cohort study highlights a significant interaction between prolonged QTc and elevated BNP levels as predictors of short-term all-cause mortality in patients with stroke-heart syndrome. Notably, patients presenting with both elevated BNP levels and prolonged QTc intervals are at a higher risk of all-cause mortality, underscoring the importance of these biomarkers in identifying high-risk individuals.

## Supplementary Materials

The Supplementary data can be found online at: www.aginganddisease.org/EN/10.14336/AD.2025.10711


