# Supplementary material for: Interaction between Brain Natriuretic Peptide and QTc Prolongation on Mortality in Patients with Stroke-Heart Syndrome
Source: Aging Dis. 2026 Jul 31;17(5):2791–803. doi: 10.14336/AD.2025.10711 (PMC13437125; doi:10.14336/AD.2025.10711)
Supplement: Supplementary file 1 [file AD-17-5-2791-s.pdf]

SUPPLEMENTARY DATA

# Interaction between Brain Natriuretic Peptide and Qtc Prolongation on Mortality in Patients with Stroke-Heart Syndrome

**Yongle Wang, Mingyi He, Lanjing Wang, Omar Elmadhoun, Fangyan Liu, Wenbo Zhao, Changhong Ren, Yuchuan Ding, Xunming Ji, Sijie Li**

© 2025. Wang Y et al. Published online at http://www.aginganddisease.org/EN/10.14336/AD.2025.10711

# SUPPLEMENTARY DATA

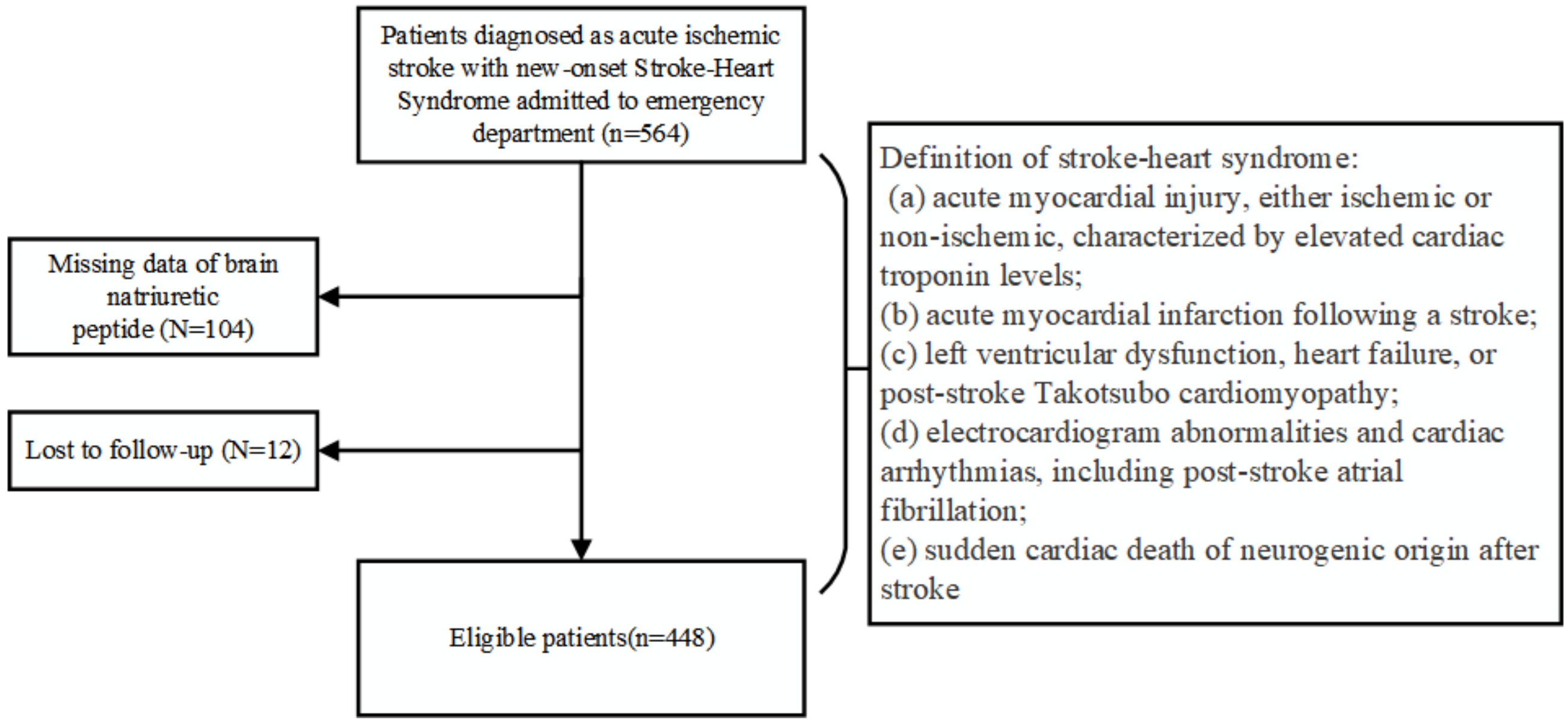


**Supplementary Figure 1. Study flowchart.** A total of 448 patients were included in final analysis.

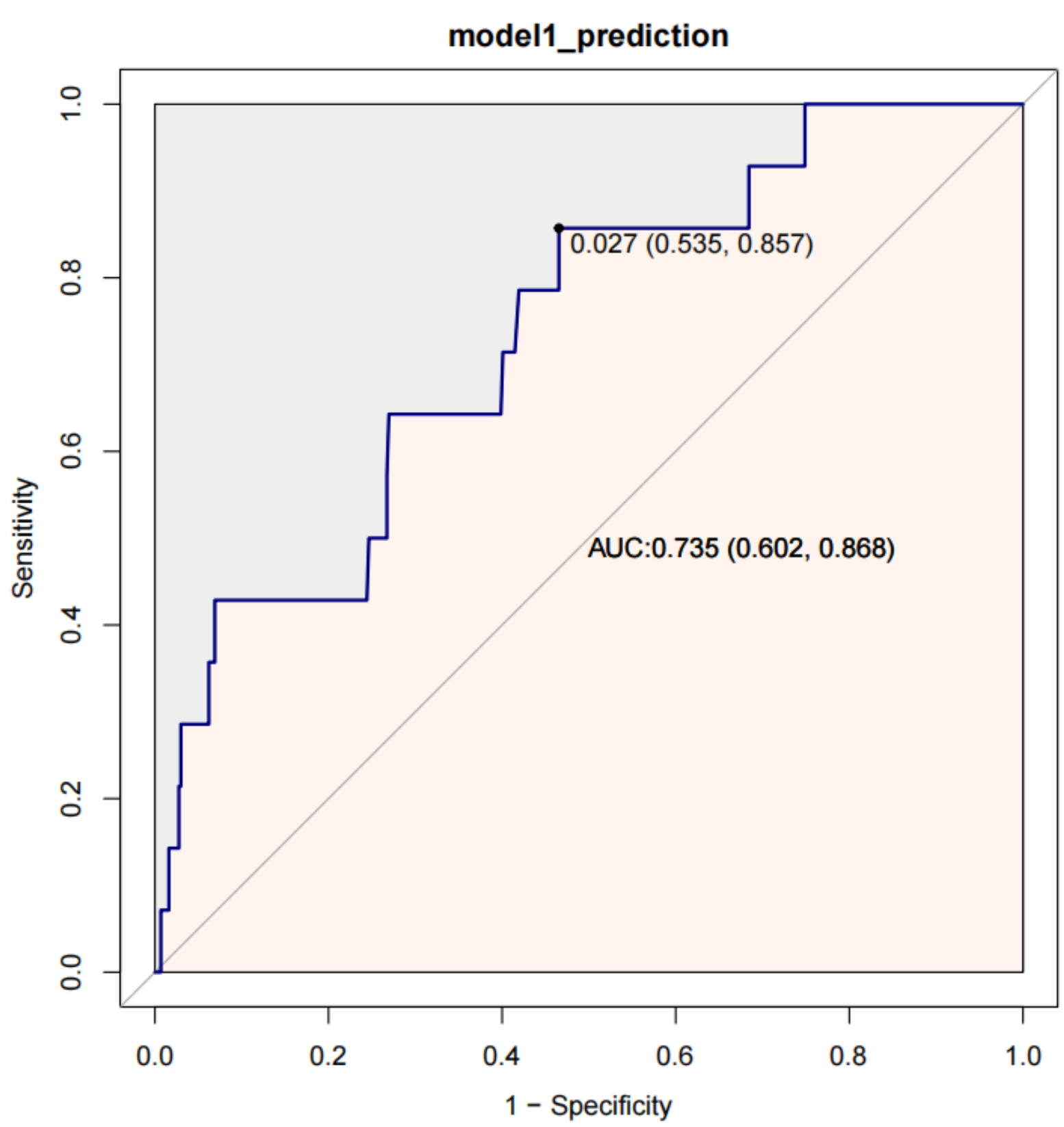


**Supplementary Figure 2. ROC plot for BNP predicting mortality.** An ROC curve determined the optimal BNP cutoff value to be 122.5 pg/mL (area under the ROC curve 0.735, sensitivity 85.7%, specificity 46.5%). This cutoff effectively distinguished patients at higher risk of mortality.

© 2025. Wang Y et al. Published online at http://www.aginganddisease.org/EN/10.14336/AD.2025.10711

**Supplementary Table 1. Univariate Cox regression analysis of risk factors associated with all-cause mortality and CCVD mortality.**

| | All-cause mortality | | CCVD mortality | |
|---|---|---|---|---|
| | HR (95%CI) | P value | HR (95%CI) | P value |
| Age, year | 1.02 (0.98,1.06) | 0.414 | 1.0027 (0.9511,1.0571) | 0.92 |
| Gender | 0.85 (0.28,2.52) | 0.765 | 1.64 (0.34,7.92) | 0.535 |
| NEWS | 1.45 (1.01,2.07) | 0.042 | 0.92 (0.5,1.71) | 0.795 |
| BNP, pg/mL | 1.05 (0.99,1.12) | 0.114 | 1.0001 (1,1.0002) | 0.292 |
| Crea, µmoI/L | 0.9996 (0.9924,1.0068) | 0.903 | 0.9977 (0.9834,1.0122) | 0.754 |
| Alb, g/L | 0.87 (0.8,0.94) | < 0.001 | 0.91 (0.81,1.01) | 0.085 |
| CK, U/L | 1.0004 (1.0001,1.0007) | 0.009 | 1.0004 (1.0001,1.0007) | 0.003 |
| Glu, mmol/L | 1.08 (1.01,1.14) | 0.014 | 1.06 (0.98,1.15) | 0.164 |
| Serum calcium, mmol/L | 0.01 (0,0.09) | < 0.001 | 0.01 (0,0.28) | 0.006 |
| Serum potassium, mmol/L | 1.22 (0.44,3.38) | 0.709 | 0.66 (0.17,2.54) | 0.546 |
| D-dimer, mg/L | 0.97 (0.82,1.15) | 0.757 | 1.1 (0.86,1.41) | 0.43 |
| QTc, per 10ms | 1.18 (1.06,1.32) | 0.003 | 1.16 (1,1.35) | 0.053 |
| Prolonged QTc | 1.93 (0.65,5.77) | 0.237 | 1.33 (0.36,4.97) | 0.668 |
| BNP≥100 pg/mL | 4.71 (1.05,21.06) | 0.042 | 2.73 (0.57,13.14) | 0.21 |
| BNP≥122.5 pg/mL | 6.72 (1.5,30.02) | 0.013 | 3.88 (0.81,18.7) | 0.091 |
| BNP per 100 pg/mL | 1.0082 (1.0023,1.0142) | 0.006 | 1.0054 (0.9954,1.0155) | 0.292 |
| DBP, mmHg | 0.9932 (0.9733,1.0134) | 0.506 | 0.9937 (0.9687,1.0194) | 0.629 |
| SBP, mmHg | 0.9951 (0.9852,1.0052) | 0.339 | 0.9939 (0.9822,1.0058) | 0.314 |
| Temperature, °C | 0.99 (0.93,1.04) | 0.668 | 0.97 (0.92,1.03) | 0.374 |
| SaO2, % | 0.9929 (0.9743,1.0119) | 0.463 | 0.99 (0.97,1.01) | 0.334 |
| HR, beats/min | 1.04 (1.02,1.07) | < 0.001 | 1.05 (1.02,1.08) | 0.001 |

Univariate Cox regression analysis identified potential risk factors for all-cause mortality within 3 months in patients with stroke-heart syndrome.

© 2025. Wang Y et al. Published online at http://www.aginganddisease.org/EN/10.14336/AD.2025.10711

**Supplementary Table 2. The interaction analysis between BNP and QTc prolongation on CCVD mortality.**

| | N.total | N.event (%) | Crude HR (95%CI) | p value | Model I HR (95%CI) | p value | Model II HR (95%CI) | p value | p for interaction |
|---|---|---|---|---|---|---|---|---|---|
| **BNP＜100 pg/mL** | | | | | | | | | 0.161 |
| QTc per 10ms | 195 | 2 (1) | 0.79 (0.43~1.44) | 0.44 | 0.81 (0.44~1.48) | 0.493 | 0.7 (0.32~1.53) | 0.371 | |
| **BNP＞=100 pg/mL** | | | | | | | | | |
| QTc per 10ms | 253 | 7 (2.8) | 1.16 (1~1.35) | 0.046 | 1.18 (1.01~1.39) | 0.038 | 1.21 (1.01~1.45) | 0.041 | |
| **BNP＜122.5 pg/mL** | | | | | | | | | 0.161 |
| QTc per 10ms | 234 | 2 (0.9) | 0.78 (0.43~1.42) | 0.418 | 0.81 (0.44~1.48) | 0.486 | 0.69 (0.3~1.58) | 0.378 | |
| **BNP＞=122.5 pg/mL** | | | | | | | | | |
| QTc per 10ms | 214 | 7 (3.3) | 1.15 (0.99~1.34) | 0.064 | 1.17 (1~1.37) | 0.057 | 1.2 (1~1.44) | 0.054 | |
| **BNP＜100 pg/mL** | | | | | | | | | 0.063 |
| Normal QTc | 107 | 2 (1.9) | 1(Ref) | | 1(Ref) | | 1(Ref) | | |
| Prolonged QTc | 88 | 0 (0) | 0 (0~Inf) | 0.999 | 0 (0~Inf) | 0.999 | 0 (0~Inf) | 0.997 | |
| **BNP＞=100 pg/mL** | | | | | | | | | |
| Normal QTc | 124 | 2 (1.6) | 1(Ref) | | 1(Ref) | | 1(Ref) | | |
| Prolonged QTc | 129 | 5 (3.9) | 2.41 (0.47~12.44) | 0.292 | 2.22 (0.42~11.7) | 0.348 | 2.3 (0.42~12.62) | 0.338 | |
| **BNP＜122.5 pg/mL** | | | | | | | | | 0.078 |
| Normal QTc | 132 | 2 (1.5) | 1(Ref) | | 1(Ref) | | 1(Ref) | | |
| Prolonged QTc | 102 | 0 (0) | 0 (0~Inf) | 0.999 | 0 (0~Inf) | 0.999 | 0 (0~Inf) | 0.997 | |
| **BNP＞=122.5 pg/mL** | | | | | | | | | |
| Normal QTc | 99 | 2 (2) | 1(Ref) | | 1(Ref) | | 1(Ref) | | |
| Prolonged QTc | 115 | 5 (4.3) | 2.16 (0.42~11.13) | 0.358 | 1.91 (0.36~10.24) | 0.451 | 2.03 (0.37~11.27) | 0.418 | |

For CCVD mortality, the interaction between QTc prolongation and BNP levels approached near significance (p = 0.063; p = 0.078).

© 2025. Wang Y et al. Published online at http://www.aginganddisease.org/EN/10.14336/AD.2025.10711
